# Indiana State Dental Association

**Published:** 1860-02

**Authors:** S. B. Smith

**Affiliations:** Indianapolis


### Proceedings of Societies.

INDIANAPOLIS, Jan. 3d, 1860, 2 o'clock, P. M.
Room of State Board of Agriculture, Capitol.

The Indiana State Dental Association met pursuant to adjournment: President, Dr. John F. Johnston, of Indianapolis, in the chair, and Dr. A. M. Moore, of Lafayette, Secretary, *pro tem.*

The members present were: Drs. T. M. Nichols, and P. G. C. Hunt, Indianapolis; Drs. John Hood, G. Lupton, H. Satterwaith, L. W. French, and J. B. Harland, Greensburg; C. C. Dills, Peru; S. B. Smith, Terre Haute; G. H. Perine, N. Y.; J. T. Toland, Cincinnati; J. P. Ulrey, Rising Sun.

The minutes of the first annual session, held December, 1858, were read and approved.

Dr. Nichols made a report as Treasurer of the Association, which was accepted.

Dr. Johnston presented the following Report, to wit:—

"Indianapolis, January 3d, 1860.

*To the members of the Indiana State Dental Association.*

"GENTLEMEN:—Having been requested by the committee representing the Pennsylvania Dental Association to call a special meeting of this society for the purpose of electing delegates to attend a preliminary m eting of delegates from other associations and colleges, to be held at Niagara Falls in August last, to advise as to the establishing of a national association on a representative basis—and the President not having the power to call a special meeting for such a purpose—and further, not deeming it advisable or necessary to do so if he had, concluded, however, as the matter seemed important, and was deemed especially so by other societies, to write each member of the Association for their views relative to the matter, requesting them, if favorable to the delegate system to write the Secretary of this Society the names of those whom they would prefer to represent us. We being entitled, according to the rule adopted by others, to three representatives, that being one to every five members. All except two responded favorably, and the following is the result of the vote as exhibited to me by the Secretary's report which I herewith hand to the society. * * *

* * * "Drs. Moore, Ulrey and Johnston having the majorities, certificates were made out and sent to them. Dr. Ulrey not being present at Niagara, I appointed Dr. Hunt to act for him, and thereby made our representation full. All of which I respectfully submit to the Association.

"JOHN F. JOHNSTON, *President.*"

Which was accepted and ordered to be put upon record.

The Committee on Order of Business then reported the following:—

1. Should pivot teeth ever be inserted; and if so, under what circumstances, and in what manner?

2. Inflamed Dentine.

3. Treatment of Dental periostitis and alveolar abscess.
4. Mechanical Dentistry, including all the various modes of constructing artificial dentures.
5. Filling teeth.
6. Miscellaneous subjects.

Which was adopted.

On motion, members of the profession present who were not members of the Association were invited to remain and participate in the discussions.

The Committee of Examination reported the names of Drs. J. T. Turner, of New London; E. W. Morris, of Waynesburg; I. Knapp, of Fort Wayne; M. H. Manlove, of Logansport, and W. C. Stanley, of Dublin; recommending them for membership, and a ballot being had they were all duly elected.

The Association then proceeded to the election of Officers for the ensuing year, which resulted in the choice of

*President.*—Dr. I. Knapp. *Vice-Presidents.*—Drs. P. G. C. Hunt, J. P. Ulrey, John Hood. *Treasurer.*—T. M. Nichols. *Rec. and Cor. Secretary.*—S. B. Smith.

The Association then adjourned to meet at 9 o'clock, A. M. to-morrow.

January 4th, 1860, 9 o'clock, A. M.

The Association met and was called to order by Dr. Johnston.

Drs. Perine and Moore were appointed to conduct the President elect to the chair. On taking the chair, the President, Dr. Knapp, made a short speech replete with the annunciation of correct principles, sentiments of devotion to the profession and enthusiastic encouragement to the members. After which Dr. Johnston read a letter from Dr. S. M. Goode, of Madison, which on motion was received and ordered to be placed on file.

The Examining Committee recommended the following for membership, viz.: Drs. S. M. Goode, of Madison; — Pifer, of Lafayette; G. A. Wells, Indianapolis; and H. T. Manlove, of Logansport; all of whom were unanimously elected.

The President announced the following Standing Committees for the current year:—

*Examining Committee.*—Drs. P. G. C. Hunt, S. B. Smith, J. P. Ulrey, M. H. Manlove, L. W. French.

*Committee on Order of Business.*—Drs. A. M. Moore, G. Lupton, J. F. Johnston.

The first subject for discussion, viz., "Pivot teeth," was taken up, several members participated, giving their views; when on motion the Association adjourned to meet at 1½ o'clock, P. M.

January 4th, 2 o'clock, P. M.

Association called to order by President Dr. Knapp.

The 2d subject for discussion, namely, "Diseased Dentine" was announced. The President, Drs. Ulrey, Johnston and Lupton gave their views. When the 3d subject, "Treatment of Dental periostitis and alveolar abscess" was taken up and discussed by Drs. Dills, Johnston, Moore, Ulrey, Hunt and Manlove, and the topic passed.

On motion the order of business was suspended to enable Dr. Lupton to exhibit casts of some interesting cases of irregularity and explain his method of treating the same.

[Which will be given in the next No. of the Register.—ED.]

Dr. Perine then exhibited several remarkable specimens of Dental exostosis and other morbid conditions.

On motion, the 4th topic in order, namely, "Mechanical Dentistry—including all the various modes of constructing artificial Dentures," was then considered, and much of interest elicited. The new "bases," "Coralite," "Amber," and "Vulcanite," received a due share of attention.

On motion it was ordered that when we finally adjourn, it be to meet in this City, at 2 o'clock, on the first Tuesday of January, 1861. Drs. Nichols and Johnston were appointed to procure a room for our next meeting.

Adjourned to 6½ o'clock, at this place.

January 4th, 1860, 6½ o'clock, P. M.

Association met. Dr. Knapp, the President, in the chair.

On motion, the regular Order of business was suspended, and the subject of a "Fee bill" was taken up and considered at some length, when Dr. Johnston moved the following resolution which was adopted. The bill also, proposed by the resolution, was adopted, after being modified as below.

*Resolved*, That the Indiana State Dental Association recommend the following *fee bill* as a fair bill of prices for the various operations therein named, and urge the propriety of our members as far as possible adopting it.

BILL OF PRICES.

| | | | |
|---|---|---|---|
| Filling smallest cavity with gold | | $2 00 | |
| " large including fang, with gold | from | 6 00 | to $25 00 |
| " with Tin | " | 1 00 | — 2 00 |
| Removing superficial decay | " | 1 00 | — 3 00 |
| " tartar | " | 1 00 | — 5 00 |
| Destroying nerve and filling fang | " | 3 00 | — 5 00 |
| " " | " | 50 | — 1 00 |
| Extracting | " | 50 | — 1 00 |
| Inserting pivot tooth | " | 3 00 | — 12 00 |
| Re-setting | " | 50 | — 1 00 |
| Riveting tooth on plate | " | 1 00 | — 1 50 |
| Soldering on tooth or clasp | " | 2 00 | — 5 00 |
| Full sett, on gold, plain, without rim | " | 100 00 | — 125 00 |
| " " gum, with rim | " | 125 00 | — 200 00 |
| " " Silver | " | 60 00 | — 70 00 |
| " " Platina | " | 125 00 | — 200 00 |
| " " Vulcanite | " | 100 00 | — 150 00 |
| " Temporary on Silver not less than | | 50 00 | |
| Single tooth on gold | " | 8 00 | — 12 00 |
| Each additional | " | 5 00 | — 6 00 |
| Regulating teeth | " | 10 00 | — 100 00 |
| Professional visit | " | 1 00 | — 3 00 |

Dr. Moore, by request of the Association, read an essay on the duties of the members of the Indiana State Dental Association, a copy of which was requested for publication.

On motion of Dr. Johnston the remaining subjects for discussion in the order of business were passed by, and the Association proceeded to the rule for miscellaneous business.

The Treasurer was instructed to correspond with delinquent members of the Association, requesting immediate payment of their dues.

On motion, the Secretary was instructed to procure 300 printed copies of the "fee bill" for the use of the Association and distribution among its members. Also a suitable book in which to record the Constitution of the Association, names of members, &c.—and draw orders on the Treasurer for the payment of the same.

Also, the President and Secretary were instructed to draw orders on the Treasurer for the amount of expenses incurred at this Session.

A ballot being ordered for the election of delegates to the Representative association, whose next meeting is to be held at Washington, D. C., in August next, which resulted in the choice of the following:

*Regular Delegates.*—Dr. M. N. Manlove, of Logansport; Drs. G. A. Wells, G. P. C. Hunt, and J. F. Johnston, Indianapolis; A. M. Moore, Lafayette; I. Knapp, Fort Wayne.

*Contingent Delegates.*—Drs. S. M. Goode, of Madison; S. B. Smith, Terre Haute; J. P. Ulrey, Rising Sun.

On motion a Committee of three members, consisting of Drs. Hunt, Wells and Moore, was appointed to audit all accounts of the Association.

Ths Secretary was instructed to take the necessary steps to secure the legal incorporation of this Association.

On motion adjourned.

S. B. Smith, *Secretary.*

